# Dynamic Alterations in the Respiratory Tract Microbiota of Patients with COVID‐19 and its Association with Microbiota in the Gut

**DOI:** 10.1002/advs.202200956

**Published:** 2022-07-03

**Authors:** Yifei Shen, Fei Yu, Dan Zhang, Qianda Zou, Mengxiao Xie, Xiao Chen, Lingjun Yuan, Bin Lou, Guoliang Xie, Ruonan Wang, Xianzhi Yang, Weizhen Chen, Qi Wang, Baihuan Feng, Yun Teng, Yuejiao Dong, Li Huang, Jiaqi Bao, Dongsheng Han, Chang Liu, Wei Wu, Xia Liu, Longjiang Fan, Michael P. Timko, Shufa Zheng, Yu Chen

**Affiliations:** ^1^ Centre of Clinical Laboratory First Affiliated Hospital College of Medicine Zhejiang University 79 Qingchun Road Hangzhou 310003 P. R. China; ^2^ Key Laboratory of Clinical In Vitro Diagnostic Techniques of Zhejiang Province Hangzhou 310003 P. R. China; ^3^ Institute of Laboratory Medicine Zhejiang University Hangzhou 310003 P. R. China; ^4^ State Key Laboratory for Diagnosis and Treatment of Infectious Diseases National Clinical Research Center for Infectious Diseases Collaborative Innovation Center for Diagnosis and Treatment of Infectious Diseases First Affiliated Hospital College of Medicine Zhejiang University Hangzhou 310003 P. R. China; ^5^ Hangzhou Global Scientific and Technological Innovation Center Zhejiang University Hangzhou 311200 P. R. China; ^6^ Department of Medical Oncology First Affiliated Hospital College of Medicine Zhejiang University Hangzhou 310003 P. R. China; ^7^ Institute of Bioinformatics Zhejiang University Hangzhou 310058 P. R. China; ^8^ Departments of Biology and Public Health Sciences University of Virginia Charlottesville VA 22904 USA

**Keywords:** coronavirus disease 2019, disease severity, gut microbiota, intensive care unit, respiratory microbiota, COVID‐19

## Abstract

The role of respiratory tract microbes and the relationship between respiratory tract and gut microbiomes in coronavirus disease 2019 (COVID‐19) remain uncertain. Here, the metagenomes of sputum and fecal samples from 66 patients with COVID‐19 at three stages of disease progression are sequenced. Respiratory tract, gut microbiome, and peripheral blood mononuclear cell (PBMC) samples are analyzed to compare the gut and respiratory tract microbiota of intensive care unit (ICU) and non‐ICU (nICU) patients and determine relationships between respiratory tract microbiome and immune response. In the respiratory tract, significantly fewer *Streptococcus*, *Actinomyces*, *Atopobium*, and *Bacteroides* are found in ICU than in nICU patients, while *Enterococcus* and *Candida* increase. In the gut, significantly fewer *Bacteroides* are found in ICU patients, while *Enterococcus* increases. Significant positive correlations exist between relative microbiota abundances in the respiratory tract and gut. Defensin‐related pathways in PBMCs are enhanced, and respiratory tract *Streptococcus* is reduced in patients with COVID‐19. A respiratory tract–gut microbiota model identifies respiratory tract *Streptococcus* and *Atopobium* as the most prominent biomarkers distinguishing between ICU and nICU patients. The findings provide insight into the respiratory tract and gut microbial dynamics during COVID‐19 progression, considering disease severity, potentially contributing to diagnosis, and treatment strategies.

## Graphical abstract

This study provides the first insights into the dynamic changes of respiratory tract and gut microbiota in the progression of coronavirus disease 2019 and disease severity. Among the respiratory tract microbiota, *Streptococcus* genera is significantly decreased in intensive care unit (ICU) patients. Significant positive correlations exist between relative microbiota abundances in the respiratory tract and gut.

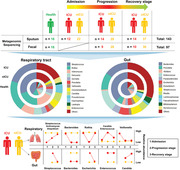

## 1 Introduction

The rapid outbreak of coronavirus disease 2019 (COVID‐19) has crippled global health and disrupted global economies. The increase in the number of severely ill patients after the spread of COVID‐19 worldwide has resulted in heavy investment in healthcare infrastructure and medical supplies. COVID‐19 is caused by severe acute respiratory syndrome coronavirus 2 (SARS‐CoV‐2), which emerged in Wuhan, China at the end of 2019.^[^ ^1^, ^2^, ^3^, ^4^ ^]^ While the majority of patients exhibit mild to moderate symptoms, up to 15% of infected individuals progress to severe pneumonia, and approximately 5% eventually develop acute respiratory distress syndrome and/or multiple organ failure.^[^ ^5^ ^]^ Higher fatality rates have been observed in elderly individuals with comorbidities and those who are immunocompromised.^[^ ^6^, ^7^, ^8^ ^]^ However, the mechanism of severe disease in patients with COVID‐19 is not well understood.

The human microbiota plays a crucial role in individual health. In particular, microorganisms residing in the gut and respiratory tract can alter susceptibility to and the outcomes of infectious diseases.^[^ ^9^ ^]^ Several studies have explored the function of the microbiome in the development of COVID‐19, suggesting possible relationships between the gut,^[^ ^10^ ^]^ nasopharyngeal,^[^ ^11^ ^]^ and oral microbiomes^[^ ^12^, ^13^ ^]^ and COVID‐19. The diversity and composition of the gut microbiota showed significant differences between patients with COVID‐19 and healthy cohorts, and these differences tended to be associated with disease severity.^[^ ^14^, ^15^ ^]^ Opportunistic bacterial and fungal pathogens were enriched in the feces of patients with COVID‐19, suggesting that secondary infections causing the dysbiosis of the gut microbiota are common in these patients.^[^ ^16^, ^17^ ^]^ The human upper respiratory tract is the major portal of entry for SARS‐CoV‐2. It contains an airway microbiome that represents its microenvironment and serves as an essential component of the airway epithelial barrier.^[^ ^18^ ^]^ However, how the respiratory tract microbiome is altered in patients with COVID‐19 is largely unknown.^[^ ^19^, ^20^ ^]^ In addition, little is known about the association between the respiratory tract microbiota and the gut microbiota, which is one of the most crucial questions regarding the contribution of the microbiota to the health of patients with COVID‐19.

The sputum microbiome can reflect microbial changes in the respiratory tract^[^ ^21^, ^22^ ^]^ and the fecal microbiome can reflect microbial changes in the gut.^[^ ^14^, ^16^ ^]^ In this study, we investigate the dynamic alterations in the respiratory tract microbiota of patients with COVID‐19 and their association with gut microbiota. We recruited 66 patients with COVID‐19 at three disease progression stages (admission, progression, and recovery) and conducted metagenome sequencing of sputum and fecal samples. A total of 143 respiratory tract microbiomes, 97 gut microbiomes, and 66 peripheral blood mononuclear cell (PBMC) transcriptomes were sequenced and subsequently used to systematically investigate the similarities and differences between the gut and respiratory tract microbiota in intensive care unit (ICU) and non‐ICU (nICU) patients at the admission, progression, and recovery stages. The findings of this study reveal the dynamic alterations in respiratory tract microbiota during COVID‐19 infection and the relationship between respiratory tract microbiota, gut microbiota, and immune responses in patients with COVID‐19. These data provide insight into the roles of changes in the respiratory tract and gut microbiomes, and immune system, at different disease progression stages and COVID‐19 severities.

## 2 Results

### 2.1 Altered Respiratory Tract and Gut Microbial Composition in Patients with COVID‐19

**Figure 1:**
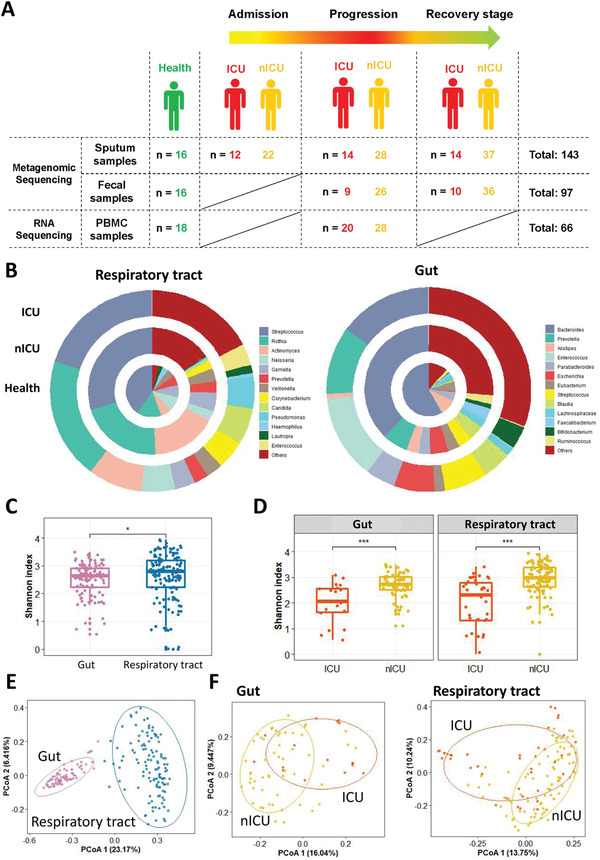
Altered respiratory tract and gut microbial compositions in patients with COVID‐19. A) Overview of the experimental design. B) Microbial compositions in the respiratory tract and gut of ICU patients (*n* = 20), nICU patients (*n* = 46), and a healthy cohort. C) Comparison of alpha‐diversity between respiratory tract and gut microbiota. D) Comparison of alpha‐diversity between the microbiota of ICU and nICU patients in the respiratory tract and gut. * *p* < 0.05, ** *p* < 0.01, *** *p* < 0.001. E) First two axes of PCoA (Bray distance) for the beta‐diversity of respiratory tract and gut microbiota. F) First two axes of PCoA (Bray distance) for the beta‐diversity of ICU and nICU patient microbiota in the respiratory tract and gut. Group differences were tested by pairwise PERMANOVA. ICU: intensive care unit; nICU: non‐ICU; PCoA: principal coordinate analysis; PERMANOVA: permutational multivariate analysis of variation; centerline, median; box limits, upper and lower quartiles; error bars, 95% CI.

To explore changes in the microbiota of patients with COVID‐19, we performed metagenome sequencing on 143 sputum and 97 fecal samples from ICU patients, nICU patients, and healthy people. Sputum samples at three disease progression stages (admission, progression, and recovery) and fecal samples at two stages (progression and recovery) were included in the study (**Figure** **1**A). *Streptococcus*, *Rothia*, and *Actinomyces* were the top three most abundant genera in both the healthy individuals and patients with COVID‐19. Interestingly, we found that the relative abundance of *Streptococcus* decreased significantly in patients with COVID‐19 compared with that in the healthy cohort (*p* < 0.01). ICU patients had a lower relative abundance of *Streptococcus* and *Actinomyces* than nICU patients (Figure 1B). Among the gut microbiota, *Bacteroides* was the most abundant genus in the healthy cohort. However, the relative abundance of *Bacteroides* decreased significantly in patients with COVID‐19 compared with that in the healthy cohort (*p* < 0.01). ICU patients had a significantly lower relative abundance of *Bacteroides* than nICU patients (*p* < 0.01). In contrast, the relative abundance of *Enterococcus* increased considerably in the fecal samples of patients with COVID‐19 (Figure 1B).

To investigate microbial diversity among patients with COVID‐19, we calculated the Shannon index to measure the alpha diversity for each patient. Alpha diversity evaluates the diversity within a sample, including richness and evenness measurements. We first compared the Shannon indices of respiratory tract and gut microbial samples. The results show that the respiratory tract microbiota had a higher Shannon index than the gut microbiota (Figure 1C). When we compared the Shannon indices of ICU and nICU patients, we observed that the ICU patients had significantly lower Shannon indices than the nICU patients in both their respiratory tract and gut microbial samples (*p* < 0.01) (Figure 1D). This finding suggests that the microbial diversity in ICU patients was significantly lower than that in nICU patients in the respiratory tract and gut (*p* < 0.01).

We further calculated beta‐diversity to evaluate differences in the microbiota among the respiratory tract and gut samples, and then combined these results using principal coordinate analysis (PCoA) dimensional reduction methods to obtain visual representations. Comparisons of beta diversity between the respiratory tract and gut samples showed that the microbial beta diversity was significantly higher in the respiratory tract than in the gut (*p* < 0.01) (Figure 1E). These results indicate that there are more differences among respiratory tract microbial compositions than among gut microbial compositions. We further compared the Shannon indices of the ICU and nICU patients for the respiratory tract and gut samples, and found that there were significant differences between the ICU and nICU patients in both the respiratory tract and gut microbiota (*p* < 0.01) (Figure 1F).

### 2.2 Dynamic Alterations in Respiratory Tract Microbiota and its Association with Disease Severity

**Figure 2:**
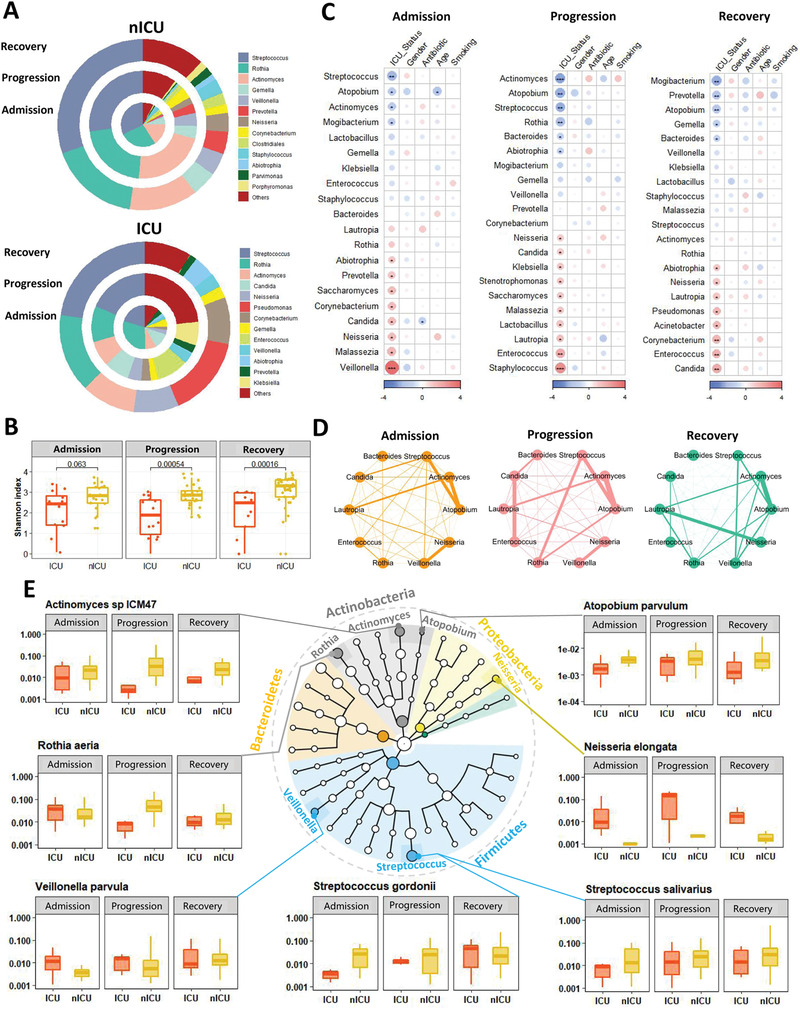
Dynamic alterations in the respiratory tract microbiota and its association with disease severity. A) Respiratory tract microbial composition of ICU patients (*n* = 20) and nICU patients (*n* = 46) at the admission, progression, and recovery stages. B) Comparison of alpha‐diversity between the respiratory tract microbiota of ICU and nICU patients at the admission, progression, and recovery stages. C) Associations between respiratory tract microbiota and patient information at the admission, progression, and recovery stages. The color in the heatmap represents the regression coefficients estimated by multiple linear model regression analyses. * *p* < 0.05, ** *p* < 0.01, *** *p* < 0.001. D) Genus correlation networks constructed for the admission, progression, and recovery stages. Edge widths are proportional to the strength of correlation. E) Genera identified in the microbiota are shown in a phylogenetic tree, grouped into the phyla Proteobacteria, Bacteroidetes, Fusobacteria, Firmicutes, and Actinobacteria. Box plots show the relative abundances of species which showed significant differences between the ICU and nICU patients at the admission, progression, and recovery stages. ICU: intensive care unit; nICU: non‐ICU; centerline, median; box limits, upper and lower quartiles; error bars, 95% CI.

We examined the relative abundance of genera in the microbiota of respiratory tract samples at the admission, progression, and recovery stages to analyze the dynamic changes in the respiratory tract microbiota. In the nICU patients, the relative abundance of *Streptococcus* was higher in the recovery stage samples than in the samples collected at the admission and progression stages (**Figure** **2**A). These results were consistent with the observation that the respiratory tract microbiota of the healthy cohort had a higher relative abundance of *Streptococcus* than that of patients with COVID‐19 (Figure 1B). In ICU patients, a higher relative abundance of *Streptococcus* was found in the recovery stage samples than in the admission and progression stage samples (Figure 2A), suggesting that the relative abundance of *Streptococcus* was associated with disease progression in both ICU and nICU patients. Moreover, *Streptococcus* abundance was significantly lower in ICU patients than in nICU patients, suggesting a significant negative correlation between *Streptococcus* relative abundance and disease severity (*p* < 0.01). Further comparison of Shannon indices between ICU and nICU patients at different stages indicated that there was a significant difference in microbiota alpha diversity between ICU and nICU patients at the progression and recovery stages (Figure 2B).

To further explore the differences in respiratory tract microbial composition between ICU and nICU patients at different stages of disease progression, we performed multiple linear model regression analyses that included information about patient ICU status, sex, age, smoking history, antibiotic usage, and corticosteroid usage. At the admission stage, the genera *Streptococcus*, *Atopobium*, *Actinomyces*, and *Mogibacterium* were significantly lower in ICU patients than in nICU patients (*p* < 0.05). In contrast, the genera *Veillonella*, *Malassezia*, *Neisseria*, and *Candida* were significantly higher in the ICU patients (*p* < 0.05) (Figure 2C). In the progression stage samples, the genera *Actinomyces*, *Atopobium*, *Streptococcus*, *Rothia*, and *Bacteroides* were significantly lower in ICU patients than in nICU patients (*p* < 0.05). In contrast, *Staphylococcus*, *Enterococcus*, *Lautropia*, *Stenotrophomonas*, *Candida*, and *Neisseria* were significantly higher in ICU patients (*p* < 0.05) (Figure 2C). In the recovery stage samples, the genera *Mogibacterium*, *Prevotella*, *Atopobium*, *Gemella*, and *Bacteroides* were significantly lower in ICU patients than in nICU patients (*p* < 0.05). In contrast, *Candida*, *Enterococcus*, *Acinetobacter*, *Pseudomonas*, *Lautropia*, and *Neisseria* were significantly higher in ICU patients (*p* < 0.05) (Figure 2C). Based on these results, we found that *Atopobium* was significantly decreased in ICU patients in all stages, whereas *Streptococcus* and *Actinomyces* decreased in the admission and progression stages. In contrast, genera previously associated with respiratory tract bacterial infections (e.g., *Enterococcus*, *Stenotrophomonas*, and *Candida*) were significantly increased in ICU patients during the progression stage (*p* < 0.05). Based on the results of the multiple linear model regression analyses, we also found that antibiotic therapy was not the cause of the differences in *Streptococcus*, *Atopobium*, *Actinomyces*, and other genera between the ICU and nICU patients during hospitalization (Figure 2C). The only difference in genus abundance was related to antibiotic *Candida* therapy in the admission stage (Figure 2C). In addition, the results show that corticosteroid usage did not have a significant influence on the abundance of *Streptococcus*, *Atopobium*, *Actinomyces*, or other genera (Figure S1, Supporting Information). Based on these findings, we suggest that the differences between the ICU and nICU patients during hospitalization were not caused by antibiotics or corticosteroid therapy.

Next, we performed a relative abundance correlation analysis to investigate the relationships between the microbial genera at each stage (Figure 2D). Our results show that *Atopobium* was significantly correlated with *Streptococcus* and *Actinomyces* in the admission, progression, and recovery stages, whereas *Neisseria* and *Veillonella* were significantly correlated with each other in the admission and progression stages (*p* < 0.05). Only *Candida* was significantly correlated with *Enterococcus* at the progressive stage (*p* < 0.05).

To determine the species involved in the observed changes in microbiota genera, we compared the relative abundances of each species between ICU and nICU patients at the admission, progression, and recovery stages (Figure 2E). In the *Streptococcus* genus, the relative abundances of three species, *Streptococcus gordonii*, *Streptococcus salivarius*, and *Streptococcus parasanguinis*, were significantly decreased in ICU patients at the admission and progression stages (*p* < 0.05). In the genus *Actinomyces*, the relative abundances of *Actinomyces oris* and *Actinomyces* sp. ICM47 decreased considerably in ICU patients at the admission, progression, and recovery stages. In the *Atopobium* genus, the relative abundance of *Atopobium parvulum* was significantly decreased in ICU patients at the admission, progression, and recovery stages (*p* < 0.05). In the *Rothia* genus, the relative abundance of *Rothia aeria* was significantly decreased in ICU patients only at the progressive stage (*p* < 0.05). In the *Veillonella* genus, the relative abundance of the species *Veillonella parvula* was significantly decreased in ICU patients only at the admission stage (*p* < 0.05). Thus, the reduced relative abundance of certain genera, such as *Streptococcus* and *Actinomyces*, was related to changes in many species, whereas in *Atopobium*, *Rothia*, and *Veillonella*, only one species was responsible for the change in the relative abundance of the genus.

### 2.3 Relationship between Respiratory Tract Microbial Composition and Clinical Indices

**Figure 3:**
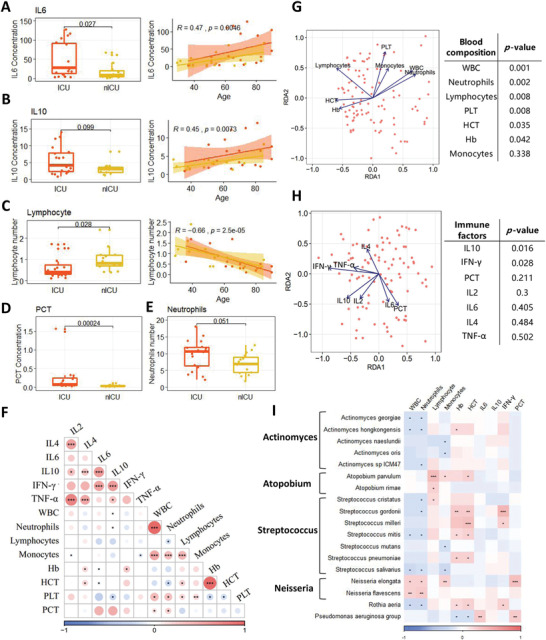
Association of patient clinical indices with respiratory tract microbiota. A) IL6. B) IL10. C) Lymphocyte number. D) PCT. E) Neutrophil number. F) Relationship between each clinical index. The color in the heatmap represents the correlation coefficients estimated by correlation analysis. * *p* < 0.05, ** *p* < 0.01, *** *p* < 0.001. G) RDA results of blood composition and microbial relative abundances. H) RDA results of immune factors and microbial relative abundances. I) Relationship between clinical indices and microbial relative abundances at the species level. The color in the heatmap represents the correlation coefficients estimated by Spearman correlation analysis. * *p* < 0.05, ** *p* < 0.01, *** *p* < 0.001. ICU: intensive care; nICU: non‐ICU; RDA: redundancy analysis; IL2: interleukin‐2, IL4: interleukin‐4, IL6: interleukin‐6, IL10: interleukin‐10, TNF‐*α*: tumor necrosis factor *α*; IFN‐*γ*: interferon *γ*; WBC: white blood cell; PLT: platelet; PCT: procalcitonin; centerline, median; box limits, upper and lower quartiles; error bars, 95% CI.

Analysis of variance (ANOVA) was performed to determine the relationships between patient age, sex, COVID‐19 severity, and clinical indices in ICU and nICU patients. We found that the levels of interleukin‐6 (IL6) and interleukin‐10 (IL10) cytokines were significantly higher in the ICU patient group than in the nICU patient group (*p* < 0.05) (**Figure** **3**A,B). The levels of IL6 and IL10 had a significant positive correlation with the age of the patients in both the ICU and nICU groups (Figure 3A,B). The lymphocyte count was significantly lower in the ICU patient group than in the nICU patient group (*p* < 0.05) (Figure 3C). This lymphopenia phenomenon has been previously reported in individuals afflicted with COVID‐19.^[^ ^23^ ^]^ The lymphocyte number was significantly negatively correlated with the age of patients in both the ICU and nICU groups (*p* < 0.05) (Figure 3C). We also found that procalcitonin (PCT) levels and neutrophil counts were significantly higher in the ICU patient group (*p* < 0.05) (Figure 3D,E).

Next, we performed a correlation analysis of the clinical indices among all the patients (Figure 3F). The white blood cell (WBC) count was significantly correlated with the number of neutrophils (*p* < 0.05). In addition, the results show that lymphocyte number was significantly negatively correlated with neutrophil number and the levels of two cytokines, IL6 and IL10. We also found that the levels of the cytokines interleukin‐2 (IL2), interleukin‐4 (IL4), IL6, IL10, tumor necrosis factor *α* (TNF‐*α*), and interferon *γ* (IFN‐*γ*) were positively correlated with each other.

To identify non‐redundant covariates of microbiome variations from correlating factors, we performed a forward stepwise redundancy analysis (RDA) among patients with COVID‐19 based on their clinical indices, including their blood composition and immune factor levels. Based on the blood composition RDA, we found that the number of neutrophils, lymphocytes, and platelets were significantly correlated with the microbial composition of patients with COVID‐19 (*p* < 0.05) (Figure 3G), and based on the immune factor RDA, we found that IL10 and IFN‐*γ* were significantly correlated with the microbial composition of these patients (*p* < 0.05) (Figure 3H).

We conducted Spearman correlation analysis between clinical indices and the relative abundances of microbial genera, to explore the relationships between the clinical indices and respiratory tract microbiota of patients with COVID‐19 (Figure 3I). The results show that neutrophil numbers were significantly negatively correlated with the relative abundances of many species of the genera *Streptococcus*, *Actinomyces*, and *Rothia* in the microbiota (*p* < 0.05). However, the relative abundance of *Neisseria* was positively correlated with neutrophil number. The lymphocyte number was significantly positively correlated with the relative abundances of species in the genus *Atopobium*, and IFN‐*γ* levels were positively correlated with the relative abundances of *Streptococcus gordonii*, *Streptococcus milleri*, and *Rothia aeria*. Interestingly, PCT levels were significantly positively correlated with *Neisseria elongate* and *Pseudomonas aeruginosa* (*p* < 0.05) (Figure 3I).

### 2.4 Defensin‐Related Pathways in PBMCs were Promoted in Patients with COVID‐19 and Associated with Respiratory Tract Microbiota

**Figure 4:**
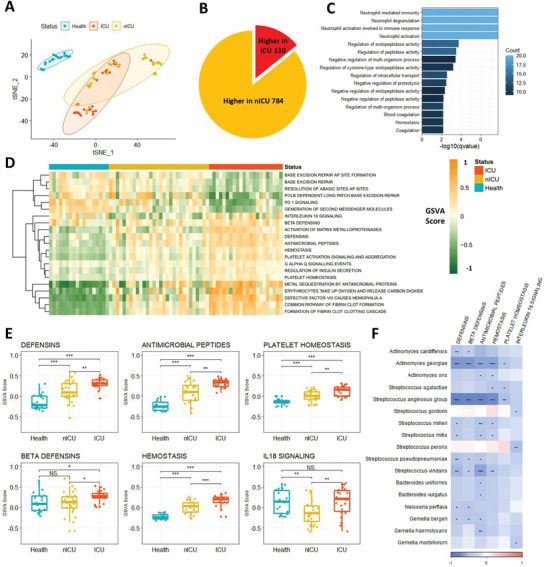
Defensin‐related pathways in PBMCs were increased in patients with COVID‐19 and associated with respiratory tract microbiota. A) Clusters of healthy donors and patients with COVID‐19 based on tSNE clustering. B) DEGs between ICU (*n* = 20) and nICU (*n* = 28) patients. C) Significantly enriched GO function terms based on up‐regulated DEGs in ICU patients. D) GSEA score of the pathways based on REACTOME database for each patient. E) Defensin‐ and hemostasis‐related pathways were up‐regulated in ICU patients compared with nICU patients F) Relationship between pathway GSEA score and microbial relative abundances at the species level. The color in the heatmap represents the correlation coefficients estimated by Spearman correlation analysis. * *p* < 0.05, ** *p* < 0.01, *** *p* < 0.001. ICU: intensive care unit; nICU: non‐ICU; tSNE: t‐distributed stochastic neighbor embedding; DEGs: differentially expressed genes; GO: gene ontology; GSEA: gene set enrichment analysis; centerline, median; box limits, upper and lower quartiles; error bars, 95% CI.

The relationships between the respiratory tract microbiota and immune response‐related gene expression were examined by RNA sequencing of PBMCs from the blood of ICU and nICU patients with COVID‐19 at the progression stage, and from 18 healthy individuals (control group). First, we performed a t‐distributed stochastic neighbor embedding (tSNE) clustering analysis based on the gene expression data of patients with COVID‐19. The tSNE clustering analysis results show that all the healthy donor samples were clustered together, while the samples of patients with COVID‐19 were separated into three clusters (**Figure** **4**A). Based on the COVID‐19 severity data, we found that among the three clusters, one contained primarily nICU patients, one contained primarily ICU patients, and the third was a mixture of nICU and ICU patients (Figure 4A). These results suggest that the ICU and nICU patients had distinct PBMC gene expression patterns.

A differentially expressed gene (DEG) analysis was performed between ICU and nICU patients with COVID‐19 to investigate which genes were related to COVID‐19 severity (Figure 4B). Among the 914 DEGs identified between the ICU and nICU patient groups, 130 genes (14.2%) were upregulated and 784 genes (85.8%) were downregulated in the ICU patient group. The results show that there were significantly more downregulated genes than upregulated genes in the ICU patients, compared to the nICU patient group (Chi‐square test *p* < 0.05).

Gene ontology (GO) enrichment analysis was performed on the DEGs that were upregulated in the ICU patients to investigate which functions were upregulated in ICU patients compared to nICU patients. “Neutrophil mediated immunity” was one of the most significantly enriched GO terms found (Figure 4C), suggesting that neutrophils were significantly activated in ICU patients (*p* < 0.05). This finding was consistent with the increased number of neutrophils in the ICU patients in the clinical index data (Figure 3E). In addition, hemostasis‐related GO terms, such as “Hemostasis” and “Blood coagulation,” were also significantly enriched in the ICU patient group (*p* < 0.05).

Gene set enrichment analysis (GSEA) based on gene expression data was then performed to systematically analyze pathway changes among the ICU, nICU, and healthy control patients. For each patient, we calculated the GSEA score of the pathways based on the REACTOME database, and based on these scores we found that the ICU patients, nICU patients, and healthy donors had distinct pathway activation patterns (Figure 4D). Interestingly, defensin‐related pathways, like “Beta defensins” and “Antimicrobial peptides,” were found to be significantly up‐regulated in patients with COVID‐19 compared to healthy individuals (*p* < 0.05). Defensin‐related pathways were also upregulated in ICU patients compared to nICU patients (Figure 4E). Moreover, we observed that hemostasis‐related pathways were upregulated in ICU patients, consistent with the results of the GO enrichment analysis. To explore the relationship between defensin‐related pathways and respiratory tract microbiota, we conducted Spearman correlation analysis between the GSEA score of each pathway and relative microbial abundances. The results show that “Defensins,” “Beta defensins,” and “Antimicrobial peptides” were all negatively correlated with the relative abundances of species in the genera *Streptococcus*, *Actinomyces*, and *Bacteroides* in the respiratory tract (Figure 4F).

### 2.5 Dynamic Alterations in the Gut Microbiota of Patients with COVID‐19 and its Association with Respiratory Tract Microbiota

**Figure 5:**
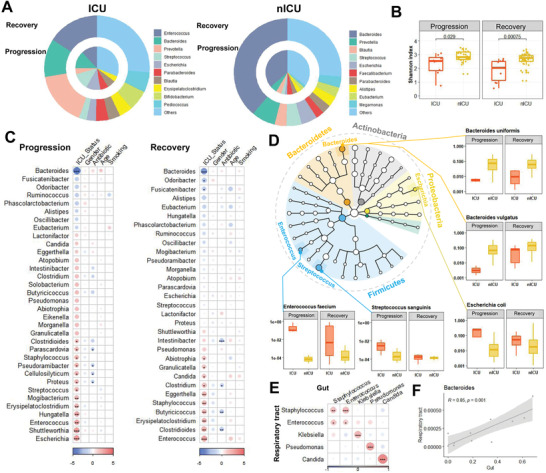
Dynamic alterations in the gut microbiota of patients with COVID‐19 and its association with respiratory tract microbiota. A) Gut microbial composition of ICU and nICU patients at the progression and recovery stages. B) Comparison of alpha‐diversity between the gut microbiota of ICU and nICU patients at the progression and recovery stages. C) Associations between the gut microbiota and patient information at the progression and recovery stages. The color in the heatmap represents the regression coefficients estimated by multiple linear model regression analyses. * *p* < 0.05, ** *p* < 0.01, *** *p* < 0.001. D) Genera identified in the microbiota are shown in a phylogenetic tree, grouped in the phyla Proteobacteria, Bacteroidetes, Fusobacteria, Firmicutes, and Actinobacteria. Box plots show the relative abundances of species which significantly changed between ICU and nICU patients at the progression and recovery stages. E) Associations between the gut and respiratory tract microbiota. The color in the heatmap represents the correlation coefficients estimated by Spearman correlation analysis. * *p* < 0.05, ** *p* < 0.01, *** *p* < 0.001. F) Correlation between the gut and respiratory tract microbiota of the *Bacteroidetes* genus. ICU: intensive care unit; nICU: non‐ICU; centerline, median; box limits, upper and lower quartiles; error bars, 95% CI.

To analyze the dynamic changes in the gut microbiota, we investigated the relative abundances of the microbiota in the gut during the progression and recovery stages. In nICU patients, the relative abundance of *Bacteroides* was higher in samples from patients in the recovery stage than in those from patients in the progression stage (**Figure** **5**A). This observation is consistent with our finding that the gut microbiota of the healthy cohort had a higher relative abundance of *Bacteroides* than that of patients with COVID‐19 (Figure 1B). In ICU patients, a higher relative abundance of *Bacteroides* was found in recovery stage samples than in progression stage samples (Figure 5A), which suggests that the relative abundance of *Bacteroides* is related to disease progression in both ICU and nICU patients. In addition, compared to the microbial composition observed in nICU patients, *Bacteroides* was significantly decreased in ICU patients, suggesting that the relative abundance of *Bacteroides* is significantly negatively correlated with disease severity (*p* < 0.05). Interestingly, *Enterococcus* rather than *Bacteroides* was the most abundant genus in ICU patients, suggesting that *Enterococcus* relative abundance was correlated with disease severity. Comparisons of the Shannon indices of ICU and nICU patients at the progression and recovery stages indicated that there was a significant difference in microbial alpha diversity between ICU and nICU patients at the progression and recovery stages (Figure 5B).

To further understand the differences in gut microbial composition between the ICU and nICU patients at different disease progression stages, we also performed a multiple linear model regression analysis that included information about patient ICU status, sex, age, smoking history, and antibiotic usage. In the progression stage samples, the genus *Bacteroides* was found to be significantly lower in ICU patients than in nICU patients (*p* < 0.05). In contrast, the genera *Escherichia*, *Enterococcus*, and *Streptococcus* were significantly increased in the ICU patients (*p* < 0.05) (Figure 5C). In the recovery stage samples, *Bacteroides* was also found to be significantly lower in ICU patients than in nICU patients. Both *Enterococcus* and *Candida* were significantly increased in the ICU patients (*p* < 0.05) (Figure 5C). Thus, *Bacteroides* was significantly decreased in ICU patients in both the progression and recovery stages, whereas genera associated with bacterial infections (such as *Enterococcus* and *Candida*) were significantly increased in ICU patients at the progression and recovery stages (*p* < 0.05).

We compared the relative abundance of each species between ICU and nICU patients at the progression and recovery stages to determine which species contributed the most to the genera‐level changes in the composition of the gut microbiota (Figure 5D). In the *Bacteroides*, the relative abundances of *Bacteroides uniformis*, *Bacteroides vulgatus*, and *Bacteroides thetaiotaomicron* were significantly decreased in ICU patients during the progression and recovery stages (*p* < 0.05). In the genus *Streptococcus*, the relative abundance of *Streptococcus sanguinis* was significantly increased in ICU patients at the progressive stage (*p* < 0.05). In the genus *Enterococcus*, the relative abundance of *Enterococcus faecium* was significantly increased in ICU patients during the progression and recovery stages (*p* < 0.05). In the *Escherichia* genus, the relative abundance of *Escherichia coli* was significantly increased in ICU patients only at the progression stage (*p* < 0.05). Thus, the decreased relative abundance of the genus *Bacteroides* could be attributed to multiple species, whereas in genera such as *Escherichia* and *Enterococcus*, only one species was correlated with the increased relative abundance of the genus.

Interestingly, we found that the relative abundances of *Enterococcus* and *Candida* were significantly increased in ICU patients in both the respiratory tract and gut microbiota (*p* < 0.05). To further investigate the relationship between the microbiota of the respiratory tract and the gut, we conducted a correlation analysis based on the relative abundances of each genus. A significant correlation was revealed between the relative abundances of infection‐related genera, such as *Enterococcus*, *Candida*, and *Pseudomonas*, in the respiratory tract and gut (Figure 5E). Species‐level investigation revealed that bacterial pathogens, such as *Enterococcus faecium*, *Candida albicans*, and *Pseudomonas aeruginosa*, were significantly correlated between the respiratory tract and gut (*p* < 0.05).

**Figure 6:**
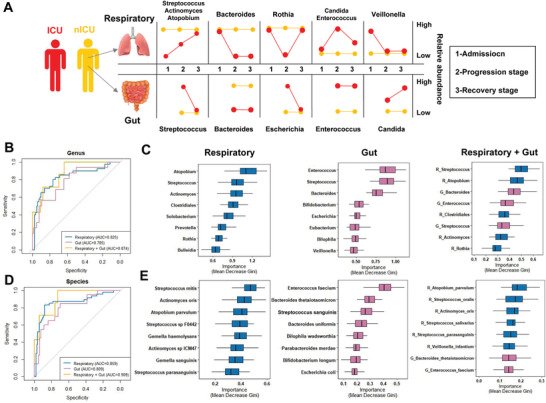
Respiratory tract and gut microbial dynamics during COVID‐19 progression and their diagnostic potential for disease severity. A) Graphical representation of major microbial alterations during disease progression in the respiratory tract and gut. ROC curves showing the discriminative ability between ICU (*n* = 20) and nICU (*n* = 46) patients using the relative abundance of the respiratory tract, gut, and combined respiratory tract–gut microbiomes at the B) genus and D) species levels. Top eight important C) genera and E) species based on Gini importance according to random‐forest classifiers based on the respiratory tract, gut, and combined respiratory tract–gut microbiomes. ICU: intensive care unit; nICU: non‐ICU; ROC: receiver operating characteristic; centerline, median; box limits, upper and lower quartiles; error bars, 95% CI.

In addition, we found that the *Bacteroides* genus was significantly reduced in ICU patients in both the respiratory tract and gut microbiota (**Figure** **6**A). The relative abundance of *Bacteroides* in the respiratory tract was significantly lower than that in the gut. Correlation analysis between the respiratory tract and gut, based on the samples in which *Bacteroides* were detected, showed a significant (*p* < 0.01) correlation between *Bacteroides* in the respiratory tract and gut (Figure 5F).

### 2.6 Respiratory Tract and Gut Microbiota Could Accurately Classify the Disease Severity of Patients with COVID‐19

To identify the microbial characteristics associated with COVID‐19 severity in the respiratory tract and gut, unsupervised random forest classification analysis using a leave‐one‐out cross‐validation method was performed at the genus and species levels. We identified microbial classifiers distinguishing ICU patients from nICU patients based on the respiratory tract, gut, and combined respiratory tract–gut microbiota in patients with COVID‐19. At the genus level, the random forest classifiers based on the respiratory tract and gut microbiota achieved area under the receiver operating characteristic curve (AUC) values of 0.825 and 0.785, respectively (Figure 6B). The combined respiratory tract–gut classifier achieved an AUC of 0.874 (Figure 6C), which was higher than the AUC values of the individual respiratory tract and gut classifiers. The random forest classifier also identified the top bacterial genera characteristics of ICU and nICU patients. For the respiratory tract classifier, *Atopobium*, *Streptococcus*, and *Actinomyces* were the top three characteristic genera. For the gut classifier, *Enterococcus*, *Streptococcus*, and *Bacteroides* were the top three characteristic genera. For the combined respiratory tract–gut classifier, *Streptococcus* and *Atopobium* in the respiratory tract and *Bacteroides* and *Enterococcus* in the gut were the top four characteristic genera between ICU and nICU patients.

At the species level, the random forest classifiers based on the respiratory tract and gut microbiota achieved AUC values of 0.859 and 0.809, respectively. The combined respiratory tract–gut classifier achieved an AUC of 0.909 (Figure 6D). Based on the results, the combined respiratory tract–gut classifier had a higher AUC than the individual respiratory tract and gut classifiers at both the genus and species levels, indicating that the combined classifier performed better in distinguishing ICU and nICU patients. Based on the random forest classifier, we also identified the top characteristic species between the ICU and nICU patients. In the respiratory tract, *Streptococcus mitis*, *Actinomyces oris*, and *Atopobium parvulum* were the top three characteristic species. In the gut, *Enterococcus faecium*, *Bacteroides thetaiotaomicron*, and *Streptococcus sanguinis*, were the top three characteristic species. In the combined respiratory tract–gut classifier, *Atopobium parvulum*, *Streptococcus oralis*, and *Actinomyces oris* were the top three characteristic species in the respiratory tract between ICU and nICU patients, and *Bacteroides thetaiotaomicron* and *Enterococcus faecium* were the seventh and eighth characteristic species in the gut between ICU and nICU patients (Figure 6E).

## 3 Discussion

The present study is among the pioneering attempts to explore the metagenomic characteristics of the respiratory tract and gut microbiota in patients with COVID‐19 at various disease progression stages and with different disease severities (ICU and nICU). We systematically examined the similarities and differences between the gut and respiratory tract microbiota in ICU and nICU patients at three stages (admission, progression, and recovery) and demonstrated the features and dynamics of the respiratory tract microbiota and its association with the gut microbiota and immune response in patients with COVID‐19. We found a significant decrease in the respiratory tract and gut microbial diversity in COVID‐19 ICU patients compared to that in nICU patients. This observation is consistent with previous studies reporting low bacterial diversity associated with respiratory viral infectious diseases.^[^ ^24^, ^25^ ^]^ However, our study also showed significant severity‐specific shifts in the overall microbial composition between COVID‐19 nICU and ICU patients, suggesting that the extent of microbiota dysbiosis might be correlated with the severity of the disease.

To analyze the dynamic changes in the respiratory tract microbiota, we first investigated the relative abundance of the microbiota in the respiratory tract at the admission, progression, and recovery stages. Our study revealed that *Streptococcus* is one of the most abundant genera inhabiting the respiratory tract in healthy individuals, consistent with previous reports.^[^ ^26^, ^27^ ^]^ We also found that three genera, *Streptococcus*, *Actinomyces*, and *Atopobium*, were significantly decreased in ICU patients compared with nICU patients at the admission and progression stages. *Streptococcus* relative abundance was significantly correlated with disease progression and severity. The depletion of *Streptococcus* might represent a dysbiotic state of the respiratory tract microbiota, possibly resulting from the introduction and overgrowth of competing microbes, or an enhanced immune response to SARS‐CoV‐2 infection.^[^ ^28^, ^29^ ^]^ The possible role of an immune imbalance is supported by the increased levels of cytokines in ICU patients and its significant correlation with microbiota variation. The increased immune response induced by viral infection could potentially alter the respiratory microbiota, and the latter can further modulate local immune responses.^[^ ^30^, ^31^ ^]^ Meanwhile, the concentration of PCT was significantly higher in ICU patients, suggesting possible co‐ and/or secondary infections in ICU patients. We suggest that both the overgrowth of competing microbes and an increased immune response to SARS‐CoV‐2 infection might play a role in the *Streptococcus* depletion in the respiratory tract microbiome. However, additional studies are needed to determine the factors that most influence disease‐severity‐related microbiome changes in COVID‐19. In addition, based on our current results, it is difficult to unequivocally conclude whether lower *Streptococcus* abundance caused changes in disease severity or whether the disease severity changes caused lower *Streptococcus* abundance. To address this question, additional studies that include more time points for sample collection before and during SARS‐CoV‐2 infection are needed.

Interestingly, we found that the genus *Veillonella* increased in ICU patients at the admission stage, and the genera *Enterococcus* and *Candida* significantly increased in ICU patients at the progression stage. The genus *Veillonella* has been shown to be a shared indicator of COVID‐19 in multiple studies,^[^ ^13^, ^32^ ^]^ and a cause of chronic anaerobic pneumonitis.^[^ ^33^ ^]^ Several members of the *Veillonella* are periodontal pathogens that are overrepresented in the bronchoalveolar lavage fluid of patients with COVID‐19.^[^ ^34^ ^]^ In addition, the genera *Enterococcus* and *Candida* have been reported to be associated with secondary bacterial infections in COVID‐19. Dysbiotic microbiota may diminish an individual's ability to resist colonization by pathogens and predispose them to secondary infection, thus leading to a poor prognosis. Our observation that microbiota with high *Streptococcus* abundances were more stable and resistant to co/secondary infection and showed lower disease severity supports this supposition.

In the present study, we found *Bacteroides* to be one of the most abundant genera in the gut microbiota of healthy individuals, and this genus was significantly lower in ICU patients than in nICU patients at the progression and recovery stages. The relative abundance of *Bacteroides* in the gut was significantly correlated with disease severity. The depletion of *Bacteroides* might represent a dysbiotic state of the gut microbiota because of the introduction and overgrowth of competing microbes. The relative abundance of the genus *Enterococcus* is known to be associated with secondary bacterial infections and it was significantly higher in the ICU patients than in the nICU patients at both the progression and recovery stages. Interestingly, we found a significant positive correlation in the relative abundance of microbiota (i.e., *Bacteroides* and *Enterococcus*) between the respiratory tract and the gut. However, whether there is a transfer of microbiota between the respiratory tract and gut remains to be determined.

Respiratory tract microbiota has been associated with susceptibility to respiratory infections and bronchiolitis, as well as with symptom severity and clinical outcomes.^[^ ^35^, ^36^ ^]^ Our results show remarkable changes in the respiratory tract microbiota between ICU and nICU patients with COVID‐19 at different stages of disease progression, indicating its potential to serve as a surrogate biomarker of COVID‐19 severity. Thus, we systematically compared the respiratory tract and gut microbiota as prognostic biomarkers for COVID‐19 severity using a random forest model. The results of our study indicate that the respiratory tract microbiota shows slightly better performance as a predictor than the gut microbiota. In addition, we found that a combined respiratory tract–gut microbiota classifier showed better performance for COVID‐19 severity prediction than either the respiratory tract or gut microbiota alone.

Our study had several limitations. First, this was a single‐center study; therefore, no evaluation of variations among patients from different locations and countries was included. Nonetheless, we are confident that our findings are representative. Second, although multiple variables (sex, age, smoking history, antibiotic usage, corticosteroid use, and sputum from coughing by conscious patients or bronchial aspirate) were considered in the microbiota analysis, dietary factors were not considered in this study. Third, although we found some similarities in the patterns of change between the respiratory tract and gut microbial composition, whether these patterns arose from a transfer of microbiota between the respiratory tract and gut is unknown, and how the microbiota might have transferred remains elusive. Finally, based on the results of this study, we could only define a correlation between microbiome changes and disease severity, and not a direct causal relationship showing that specific microbial changes or changes in genera abundance lead to changes in disease severity.

## 4 Conclusion

In summary, the present study shows that dynamic changes occur in the respiratory tract and gut microbial communities of patients with different COVID‐19 disease severities, and that significant changes in the composition of the respiratory tract and gut microbiomes can be found between ICU and nICU patients. Our findings demonstrate that the respiratory tract microbial composition of patients with COVID‐19—particularly changes in the abundances of three genera, *Streptococcus*, *Atopobium*, and *Actinomyces*—could be used as a noninvasive biomarker for dysbiosis of the pulmonary microbiome or the invasion of potential pathogens in the lungs. Second, these studies provide substantial evidence that major potential pathogens (*Enterococcus* and *Candida* genera) are associated with lung co‐infections in COVID‐19, and thus guide antibiotic treatment of secondary bacterial infections in COVID‐19. Finally, the transcriptome sequencing data provided here conclusively show that defensin‐related pathways in PBMCs increase in patients with COVID‐19, and these changes are associated with changes in the microbial composition of the respiratory tract. Taken together, our study provides new and important data that will be valuable for halting the continuing and insidious COVID‐19 health crisis.

## 5 Experimental Section

### Study Design and Participants

Sixty‐six patients with COVID‐19 (46 nICU hospitalized patients and 20 ICU hospitalized patients) and 18 healthy individuals from the First Affiliated Hospital of Zhejiang University were included in this study. This cohort covered a wide age range, in which older patients constituted a greater fraction of the severe symptom group (Table S1, Supporting Information), consistent with previous reports on the age and severity distribution of the disease.^[^ ^8^ ^]^ The ICU patients met one of the following conditions: 1) respiratory failure and required mechanical ventilation, 2) shock, and 3) complicating nonfunction of other organs.^[^ ^37^ ^]^ These patients were included in the ICU group. Other hospitalized patients were included in the nICU group. Sputum and fecal samples were collected from patients at different disease stages (admission, progression, and recovery). The disease stages of the patients were determined by a team of physicians based on clinical symptoms, chest CT scans, laboratory indicators, and virological test results. Patients in the progression stage were identified by the progression of pulmonary inflammatory lesions compared with those in the admission stage and had high levels of laboratory inflammation indicators. Patients in the recovery stage were identified by the improvement of clinical symptoms and respiratory function, absorption of pulmonary inflammation, and negative virological test results. In addition, PBMC samples were collected from 48 patients (28 in the nICU and 20 in the ICU) in the progression stages. PBMC RNA‐seq data was collected from all 18 healthy donors, and sputum and fecal microbiome sequencing data from 16 of them. The study protocol was approved by the Ethics Committee of the First Affiliated Hospital, Zhejiang University School of Medicine, China (2021IIT A0239).

Epidemiological, clinical, and laboratory characteristics, as well as treatment and outcome data, were obtained through data collection from hospital electronic medical records. A trained team of doctors reviewed the data. The clinical data included personal characteristics, comorbidities, date of symptom onset, symptoms and signs, laboratory indicators, timing of antiviral treatment, and the progression and resolution of clinical illness. IL2, IL4, IL6, IL10, IFN‐*γ*, TNF‐*α*, and PCT levels and lymphocyte, neutrophil, and monocyte numbers were included as laboratory indicators. The documented comorbidities included diabetes mellitus, heart disease, chronic lung disease, renal failure, liver disease, HIV infection, cancer, and immunosuppressive treatment, including corticosteroids. The symptoms started when fever, cough, chills, dizziness, headache, or fatigue appeared.

### Sample Collection

The respiratory tract samples included in this study were sputum from conscious patients and bronchial aspirates from unconscious patients. The detailed method for sputum sample collection is described in the Supporting Information section. Blood samples were collected in special whole‐blood collection tubes. PBMCs were isolated using standard density gradient centrifugation and used for RNA‐seq analysis. The fecal samples were collected in the hospital with a special sterile container, stored frozen on dry ice, incubated in Dulbecco's phosphate‐buffered saline with agitation for 15 min, and filtered through 40‐micron filters. All medical staffs were equipped with personal protection equipment for biosafety level 3 during sampling, including solid front‐wraparound gowns, goggles, and N95 respirators.

### Library Generation for PBMC Transcriptome Sequencing

Total RNA was isolated from the PBMC samples using the QIAamp RNA Blood Mini Kit (Qiagen, Valencia, CA, USA) according to the manufacturer's instructions. Total RNA (1 µg) was used to prepare a sequencing library. PolyA‐tailed RNAs were selected using mRNA capture beads (VAHTS) and the Illumina Total RNA‐seq (H/M/R) Library Prep Kit was used according to the manufacturer's instructions. Library quality was examined using an Agilent Bioanalyzer 2100. The libraries were pooled in equimolar amounts to a final concentration of 2 nm. The normalized libraries were denatured with 0.1 m NaOH (Sigma) and the pooled denatured libraries were pair‐end sequenced with a 150‐bp read length in the Illumina NovaSeq 6000 platform.

### Library Generation for Metagenome Sequencing

DNA was extracted from frozen sputum and fecal samples using a TIANamp Micro DNA Kit (DP316, Tiangen Biotech) according to the manufacturer's recommendations, after the host cells were removed using a self‐developed host‐removal kit. DNA quality was assessed using an Agilent 4200 TapeStation (Agilent Technologies). DNA libraries were constructed using standardized protocols for DNA fragmentation, end repair, adapter ligation, and PCR amplification. Library quality was assessed using an Agilent Bioanalyzer 2100. Whole‐genome shotgun sequencing of the sputum and fecal samples was performed on an Illumina HiSeq2500 platform. All samples were paired‐end sequenced with a 150‐bp read length to a targeted data size of 5.0 Gb.

### RNA‐seq Data Process

FASTQC^[^ ^38^ ^]^ was used to determine the initial read quality control (QC) metric (base quality distribution). NGS QC toolkits^[^ ^39^ ^]^ were used to trim the low‐quality reads. The RNA‐seq reads were mapped to the reference genome using Tophat.^[^ ^40^ ^]^ Following alignment, Cufflinks^[^ ^40^ ^]^ was used for transcript assembly. Transcripts generated from all the sequencing samples were merged using Cuffmerge^[^ ^40^ ^]^ and the output files were imported separately into Cuffdiff^[^ ^40^ ^]^ for further statistical analysis.

### Metagenome Data Process

Sequence reads were passed through the KneadData QC pipeline (http://huttenhower.sph.harvard.edu/kneaddata), which incorporates the Trimmomatic^[^ ^41^ ^]^ and BMTagger^[^ ^42^ ^]^ filtering and decontamination algorithms to remove low‐quality read bases and reads of human origin, respectively. Trimmed non‐human reads shorter than 50 nucleotides were discarded. Samples with < 500 000 microbial reads were excluded. Taxonomic profiling was performed using the MetaPhlAn2 classifier.^[^ ^43^ ^]^ The classifier relied on approximately 1 million clade‐specific marker genes derived from > 10 000 microbial genomes to unambiguously classify reads to taxonomies and yield the relative abundances of the taxa identified in the sample. To analyze the microbial differences in COVID‐19 severity, multiple linear model regression analysis^[^ ^44^ ^]^ (including patient information) and linear discriminant analysis effect size analysis^[^ ^45^ ^]^ were also performed to identify microbiota biomarkers between groups of different COVID‐19 severity. In addition, to perform taxonomic classifications of all the reads, Kraken2 was used to perform exact *k*‐mer matching to sequences within the NCBI non‐redundant database, using lowest common ancestor algorithms.

### Identification of Microbiota Markers for Predicting COVID‐19 Severity Based on Respiratory Tract and Gut Metagenome Data

To identify microbial markers that distinguish samples from ICU and nICU patients, classification models were constructed based on genus and species profiles using random‐forest models. The models were validated by 10‐fold stratified cross‐validation testing (i.e., the dataset partitions were resampled 10 times). In each test, the accuracy of the model was examined using the receiver operating characteristic, and abundance filtering was performed to remove low‐abundance features by calculating the average relative abundance. Two steps were performed in the random‐forest model. In the first step, the model was constructed using each of the two microbial profiles (respiratory tract and gut) independently. All the prefiltered features were used to perform a random‐forest function with the indicated parameters (500 trees, balanced class weight) and compute the “feature importance” (Mean Decrease Gini). The optimal number of features was determined using the recursive feature elimination method^[^ ^46^ ^]^ with a parameter step  =  0.1 and five different random seeds. In the second step, the combination model was constructed using features determined from the separate respiratory tract and gut models. The features were then selected using the recursive feature elimination method.^[^ ^46^ ^]^ All analyses were performed using the R software (ver. 4.0.0) package, “randomForest”.

### Statistical Analysis

DEGs were identified using a significance threshold of *q* value (false discovery rate) ≤ 0.05. R software (ver. 4.0.0) was used for tSNE to cluster the data, explore the relationships between different samples, and identify outliers. GO enrichment analysis was implemented using the “clusterProfiler” R package.^[^ ^47^ ^]^ GO terms with a corrected FDR ≤ 0.05 were considered significantly enriched. Single‐sample GSEA was used to evaluate the enrichment score of each sample. It was conducted using the R package “GSVA”.^[^ ^48^ ^]^ REACTOME gene sets were downloaded from the Broad Institute website (https://www.gsea‐msigdb.org/gsea/).

Alpha diversity was used to evaluate the diversity within a sample, including richness and evenness measurements. The R software (ver. 4.0.0) package “vegan”^[^ ^49^ ^]^ was used to calculate the alpha diversity (Shannon index) among the patients with COVID‐19. The differences in alpha diversity among and between groups were statistically evaluated using permutational multivariate ANOVA or the permutation test with a *p*‐value calculated based on 2000 permutations. Fisher's exact test and the Mann–Whitney *U* test were used to compare categorical and continuous variables, respectively. Multiple comparisons were corrected using the false discovery rate algorithm with a cut‐off value of 0.05. Beta diversity was used to evaluate differences in the microbiomes among the samples. PCoA‐dimensional reduction methods were also used to obtain visual representations. This analysis was implemented in the R “vegan” package^[^ ^49^ ^]^ and visualized using scatter plots.

RDA was performed among the patients with COVID‐19 based on clinical indices to identify non‐redundant covariates of microbiome variations from correlating factors. RDA extracted and summarized the variations in a set of response variables explained by a set of explanatory variables. Correlation analysis was used to reveal associations between taxa and sample metadata. Network analysis was used to explore the co‐occurrence of microbial taxa in patients with COVID‐19 based on Spearman correlation analysis. The correlation networks represented potential interactions between co‐occurring microbial taxa. Correlation coefficients and significant *p*‐values were computed using R software. The networks were visualized and analyzed using Cytoscape.^[^ ^50^ ^]^

## Conflict of Interest

The authors declare no conflict of interest.

## Author Contributions

Y.S., F.Y., D.Z., and Q.Z. contributed equally to this work. Y.C. and S.Z. contributed equally to this study and are joint corresponding authors. Y.C. and S.Z. were the co‐principal investigators, designed, and supervised the study, and wrote the grant application (assisted by Y.S.). S.Z., F.Y., D.Z., Q.Z., M.X., L.Y., B.L., G.X., X.Y., W.C., Q.W., B.F., Y.T., Y.D., L.H., and W.W. were involved in the recruitment, data collection, and clinical management. Y.S., S.Z., and Y.C. performed bioinformatics, statistical analysis, and data interpretation. Y.S., S.Z., Y.C., X.C., M.T., J.B., D.H., C.L., X.L., and W.W. contributed to the discussion of the results. Y.S., S.Z., Y.C., M.T., and L.F. drafted the article. All authors contributed to the review and revision and have seen and approved the final version. The corresponding author attests that all listed authors meet the authorship criteria and that no others meeting the criteria have been omitted.

## Supporting information

Supporting Information

## Data Availability

The data that support the findings of this study are available on request from the corresponding author. The data are not publicly available due to privacy or ethical restrictions.
