# Supplementary material for: Dynamic Alterations in the Respiratory Tract Microbiota of Patients with COVID‐19 and its Association with Microbiota in the Gut
Source: Adv Sci (Weinh). 2022 Jul 3;9(27):2200956. doi: 10.1002/advs.202200956 (PMC9350109; doi:10.1002/advs.202200956)
Supplement: Supplementary file 1 — Supporting Information [file ADVS-9-2200956-s001.pdf]

ADVANCED SCIENCE
Open Access

Supporting Information

for *Adv. Sci.*, DOI 10.1002/advs.202200956

Dynamic Alterations in the Respiratory Tract Microbiota of Patients with COVID-19 and its Association with Microbiota in the Gut

Yifei Shen, Fei Yu, Dan Zhang, Qianda Zou, Mengxiao Xie, Xiao Chen, Lingjun Yuan, Bin Lou, Guoliang Xie, Ruonan Wang, Xianzhi Yang, Weizhen Chen, Qi Wang, Baihuan Feng, Yun Teng, Yuejiao Dong, Li Huang, Jiaqi Bao, Dongsheng Han, Chang Liu, Wei Wu, Xia Liu, Longjiang Fan, Michael P. Timko, Shufa Zheng and Yu Chen**

Supplementary Method

Method for coughed sputum samples collection:

A well-trained nurse was responsible for collection of the sputum samples using clinical protocol based on the "Manual of Clinical Microbiology 12th edition" [1]. Before coughed sputum sampling, it was confirmed that the patient did not drink water, eat food within half an hour. To reduce the oral contamination, subjects were asked to clear their throat and rinse their mouth with water. Then the patients were asked to deep cough 3–5 times before spitting sputum into a sterile container. We will not use the unqualified samples if we are not able to collect the sputum or the sputum contained much saliva.

Method for bronchial aspirate sputum samples collection:

1. Washing hands and wear facemask
2. Equipment preparation: Stethoscope, Oxygen, Flow meter, Respiratory capsule, Oxygen connecting pipe, Disposable treatment bowl, Normal saline, Sputum diluent, Furacilin Solution, Disposable sputum suction tube, Negative pressure suction device.
3. Assessment: Signs of excessive sputum: secretions in the tracheal tube, audible sputum sounds in lung auscultation, airway high pressure alarm, low tidal volume alarm, decreased oxygen saturation and too fast respiratory rate were directly observed.
4. Preparation for sputum samples collection: **A.** Press the pure oxygen button of the ventilator to inhale for 1 ~ 2 minutes or pressurize the pure oxygen with the breathing skin bag for 10 ~ 15 times (or extend the time according to the patient's condition) **B.** Start the aspirator, adjust the pressure and confirm that the pressure is normal. **C.** Open two disposable treatment bowls (open them from the bottom of the package and do not pollute the inner surface of the treatment bowl) and pour normal saline respectively. **D.** Open the outer package of the sputum suction tube, expose the end, wear gloves, keep one hand sterile, and take out the sputum suction tube. Connect the connector of the sputum suction pipe with the negative pressure suction pipe, adjust the pressure to 100 ~ 120mmhg (13.3kpa), and the maximum pressure shall not exceed 200 mmHg (26.7kpa), and try suction.
5. Sputum samples collection: **A.** Gently insert the sputum suction tube into the endotracheal

tube (do not under negative pressure). **B.** Determine the insertion depth of the sputum suction tube (if one item is met): the depth of the sputum suction tube is close to the length of the endotracheal tube; The patient has cough reflex; Under normal circumstances, the sputum suction tube can no longer go deep; Patients with lobectomy can refer to the surgeon's advice. **C.** Intermittent suction: rotate the sputum suction tube with your index finger and thumb, lift it while sucking, and stay in many places to improve the sputum suction efficiency. Do not lift and insert the sputum suction tube up and down, and the suction time should not exceed 15 seconds. When the patient has decreased oxygen saturation or dyspnea, stop suction immediately. **D.** Press the pure oxygen button of the ventilator to inhale for 1 ~ 2 minutes, or pressurize the pure oxygen with the breathing skin bag for 10 ~ 15 times (or extend the time according to the patient's condition), and then inhale again. **E.** After the sputum suction tube is taken out, draw normal saline and flush the sputum in the tube to avoid obstruction. **Note**: Strictly aseptic operation. It is strictly prohibited to perform endotracheal suction after oral or nasal suction. Closely monitor heart rate, blood pressure, respiration and oxygen saturation during sputum suction to avoid hypoxia.

6. Record: The changes of respiratory sound, secretion clearance, respiratory morphology and patient response before and after sputum suction.

Both of the coughed and the bronchial aspirate sputum samples were evaluated by experienced specialists before the samples included the study. The specialists will microscopically evaluate the sputum samples quality based on the presence of squamous epithelial cells (SECs), inflammatory cells, and bacteria [1].

Reference

1. Karen C C, Michael A P, Marie L L, et al. Manual of clinical microbiology: Volume 1[M]. ASM press, 2019.

Supporting Information

Dynamic Alterations in the Respiratory Tract Microbiota of Patients with COVID-19 and its Association with Microbiota in the Gut

Yifei Shen, Fei Yu, Dan Zhang, Qianda Zou, Mengxiao Xie, Xiao Chen, Lingjun Yuan, Bin Lou, Guoliang Xie, Ruonan Wang, Xianzhi Yang, Weizhen Chen, Qi Wang, Baihuan Feng, Yun Teng, Yuejiao Dong, Li Huang, Jiaqi Bao, Dongsheng Han, Chang Liu, Wei Wu, Xia Liu, Longjiang Fan, Michael P. Timko, Shufa Zheng*, and Yu Chen*

Figure S1. The associations between the respiratory tract microbiota and patient information at admission, progression, and recovery stages. The color in the heatmap represents the regression coefficients estimated by multiple linear model regression analyses. * $p < 0.05$, ** $p < 0.01$, *** $p < 0.001$. (Sample_type: Coughed sputum or Bronchial aspirate sputum samples)

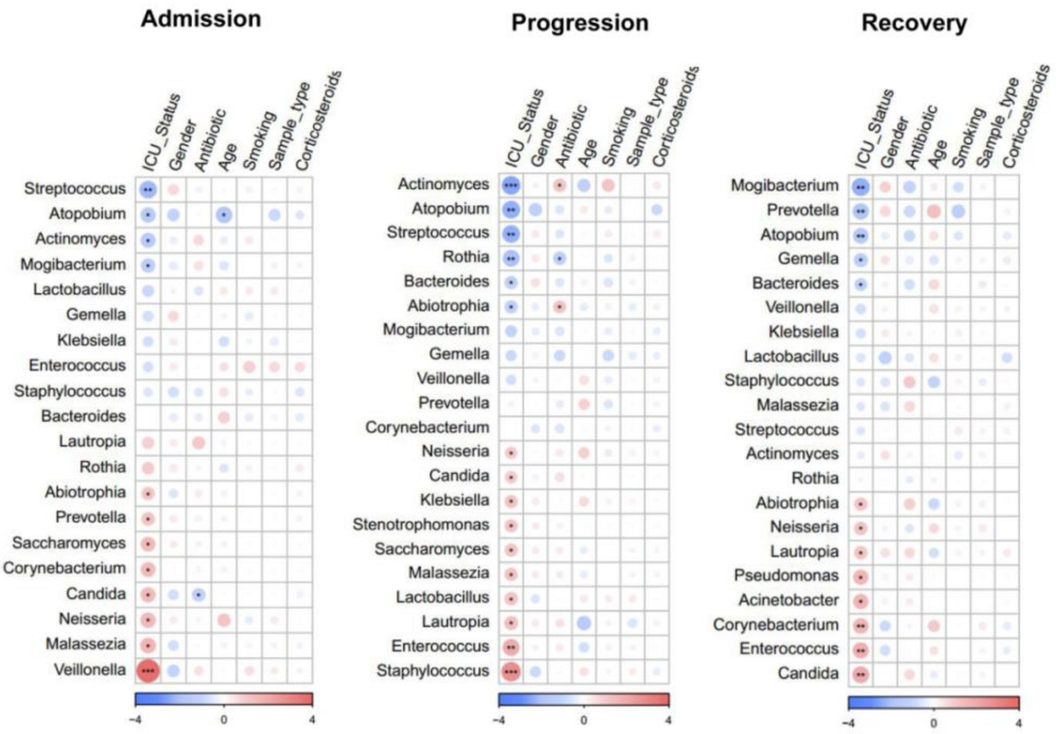

Table S1. Basic demographics of the patients used in this study.

Characteristic	No ICU (n = 46)	ICU (n = 20)	*P* values
**Age, y**	51 (36.8–57.5)	70 (55.3–83.0)	**<0.001**
**Male sex**	26 (56.5)	16 (80)	0.068
**Smoking history**	7 (15.2)	2 (10)	0.712
**Comorbidities**			
Any coexisting comorbidity	11 (23.9)	15 (75)	**<0.001**
Hypertension	7 (15.2)	13 (65)	**<0.001**
Diabetes	4 (8.7)	4 (20)	0.232
Cardiac disease	2 (4.3)	3 (15)	0.159
Liver disease	1 (2.2)	1 (5)	0.517
Lung disease	0 (0)	3 (15)	**0.025**
Immune compromise	0 (0)	1 (5)	0.303
**Signs and Symptoms**			
Fever	39 (84.8)	18 (90)	0.570
Cough	28 (60.9)	10 (50)	0.412
Sputum	12 (26.1)	7 (35)	0.462
Dizziness	5 (10.9)	1 (5)	0.659
Chest distress	4 (8.7)	2 (10)	1.000
Headache	4 (8.7)	0 (0)	0.306
Gastrointestinal symptoms	3 (6.5)	7 (35)	**0.006**
Fatigue	3 (6.5)	5 (25)	**0.049**
**Laboratory results**			
Leukocyte count, mm^3	3.1 (1.9–5.9)	6.7 (3.6–12.6)	**<0.001**
Lymphocyte count, mm^3	1.0 (0.7–1.3)	0.5 (0.4–0.8)	**<0.001**
Platelet count, mm^3	181 (130–233)	182 (141.8–195.5)	0.639
Fibrin, g/L	4.2 (3.4–4.8)	4.6 (4.3–5.5)	**0.008**
D-dimer, ug/lFEU	267 (85.0–442.5)	448 (296–953)	**0.009**
Albumin, g/L	42.1 (38.3–45.3)	34.3 (30.3–39.9)	**<0.001**
Aspartate transaminase, IU/L	20.5 (18.0–31.8)	28.5 (22.8–42.8)	**0.029**
Creatinine, µmol/L	76 (61–88)	90.5 (70.0–106.8)	**0.009**
Creatine kinase, IU/L	77.5 (52–120)	95.5 (61.0–266.5)	0.132
Lactate dehydrogenase, IU/L	227.5 (191.5–297.8)	319.5 (244–382)	**0.006**
C-reactive protein, mg/L	16.2 (8.6–35.5)	51.2 (24.2–87.3)	**<0.001**
Procalcitonin, ng/mL	0.05 (0.03–0.07)	0.09 (0.05–0.4)	**0.003**
Interleukin-2, pg/mL	0.5 (0.0–1.3)	0.5 (0.4–1.0)	0.601
Interleukin-4, pg/mL	0.9 (0.7–0.9)	0.9 (0.7–0.9)	0.825
Interleukin-6, pg/mL	17.3 (7.0–47.5)	46.6 (24.0–83.3)	**0.016**
Interleukin-10, pg/mL	3.7 (2.9–7.7)	6.9 (4.3–8.9)	0.117
$CD4^+$ lymphocytes, µL	376 (214–514)	106.5 (71.3–209.5)	**<0.001**
$CD8^+$ lymphocytes, µL	278 (166–446)	79 (68.3–129.0)	**<0.001**
$CD19^+$ lymphocytes, µL	167 (91–212)	56.5 (27.0–128.5)	**0.007**
$CD56^+$ lymphocytes, µL	176 (85–266)	84.5 (50.3–154.4)	**0.021**

**Treatments and outcomes**			
Antiviral treatment	46 (100)	20 (100)	**-**
Antibiotic treatment	10 (21.7)	18 (90)	**<0.001**
Gamma globulin	21 (45.7)	16 (80)	**0.010**
Glucocorticoids	33 (71.7)	20 (100)	**0.006**
Invasive mechanical ventilation	0 (0)	9 (45)	**<0.001**
ECMO	0 (0)	5 (25)	**0.002**
CRRT	0 (0)	1 (5)	0.303
Discharged	46 (100)	20 (100)	**-**
